# Gadolinium-Doped Gallic Acid-Zinc/Aluminium-Layered Double Hydroxide/Gold Theranostic Nanoparticles for a Bimodal Magnetic Resonance Imaging and Drug Delivery System

**DOI:** 10.3390/nano7090244

**Published:** 2017-08-31

**Authors:** Muhammad Sani Usman, Mohd Zobir Hussein, Sharida Fakurazi, Mas Jaffri Masarudin, Fathinul Fikri Ahmad Saad

**Affiliations:** 1 Materials Synthesis and Characterization Laboratory, Institute of Advanced Technology (ITMA), Serdang 43400, Selangor, Malaysia; 2 Laboratory of Vaccines and Immunotherapeutics, Institute of Bioscience, Serdang 43400, Selangor, Malaysia; sharida.fakurazi@gmail.com; 3 Department of Human Anatomy, Faculty of Medicine and Health Sciences, Serdang 43400, Selangor, Malaysia; 4 Department of Cell & Molecular Biology, Faculty of Biotechnology and Biomolecular Sciences, Serdang 43400, Selangor, Malaysia; masjaffri@upm.edu.my; 5 Centre for Diagnostic and Nuclear Imaging, Faculty of Medicine and Health Sciences, Universiti Putra Malaysia, Serdang 43400, Selangor, Malaysia; ahmadsaadff@gmail.com

**Keywords:** Zn/Al LDH, gallic acid, therapeutic, diagnostic, theranostic

## Abstract

We have developed gadolinium-based theranostic nanoparticles for co-delivery of drug and magnetic resonance imaging (MRI) contrast agent using Zn/Al-layered double hydroxide as the nanocarrier platform, a naturally occurring phenolic compound, gallic acid (GA) as therapeutic agent, and Gd(NO_3_)_3_ as diagnostic agent. Gold nanoparticles (AuNPs) were grown on the system to support the contrast for MRI imaging. The nanoparticles were characterized using techniques such as Hi-TEM, XRD, ICP-ES. Kinetic release study of the GA from the nanoparticles showed about 70% of GA was released over a period of 72 h. The in vitro cell viability test for the nanoparticles showed relatively low toxicity to human cell lines (3T3) and improved toxicity on cancerous cell lines (HepG2). A preliminary contrast property test of the nanoparticles, tested on a 3 Tesla MRI machine at various concentrations of GAGZAu and water (as a reference) indicates that the nanoparticles have a promising dual diagnostic and therapeutic features to further develop a better future for clinical remedy for cancer treatment.

## 1. Introduction

Nano-based materials or nanotechnology research has become a hot zone of material science research globally, due to the various advantages that come with nanomaterials [1], especially in numerous biomedical applications [2,3,4,5,6]. Chemotherapy remains the best choice of cancer treatment, due the availability of various anticancer agents. Nevertheless, the challenge of toxicities posed by these agents still exits [7]. Over a decade or so, various nanotechnology platforms have been explored in overcoming this challenge. Some of the areas of interest are the nanodrug delivery systems (NDDS). However, multimodal delivery systems (MDS) are recently gaining more attention [8], where drugs are simultaneously loaded along with other active agents on the same nanocarrier platform [7,9]. For instance, in theranostic research, the concept of MDS is employed where a nanocarrier is used as a delivery agent for therapeutic agents and diagnostic agents, such as magnetic resonance imaging (MRI) contrast agents [6], for a non-invasive concurrent delivery [10].

MRI is a non-invasive and non–ionizing important imaging tool used for cancer clinical diagnosis. Since the 1970s, MRI has been one of the most recognized powerful imaging techniques due to its high spatial resolution and tissue penetrating ability [8,11]. However, the use of MRI often requires contrast agents. Gadolinium-based (Gd) contrast agents are the commonly used contrast agents for MRI, which improve the T1 and T2 relaxation times of the images produced [8]. T1 and T2 relaxations are the main signals among others generated during MR imaging, which have distinctive grey-scale color contrasts reflecting the fluid and soft tissue composition of human subject. The signals represent spin–lattice relaxation and spin–spin relaxation for T1 and T2, respectively [12]. Zn/Al-layered double hydroxide (LDH) is one of the candidates capable of simultaneously intercalating and adsorbing theranostic agents due to their exchangeable interlayer anions. LDH is one of the group of two-dimensional layered structures materials [13] and has the general formula of [$M_{1-x}^{2+}$ $M_{x\;}^{3+}$ (OH)_2_]*_x_* + [A*^n^*^−^] *x*/*n*·*m*H_2_O] [14], where divalent and trivalent metal cations are represented by M^2+^ and M^3+^, respectively, and interlayer exchangeable anions are represented by [A*^n^*^−^], and water as *x*/*n* [8,15,16,17]. Gallic acid is the therapeutic agent employed in this research; it is a naturally occurring polyhydroxyl phenolic compound, often found in different kinds of fruits. It is believed to have anticancer properties as well as other activities in a range of cells [18]. Although there are various research publications on drug intercalation using LDH in drug delivery as reviewed by Kura et al. [19], only a few works have so far been done on theranostic applications using LDH-based nanocarriers. Those articles have also been reviewed by Usman et al. [8], amongst which none has reported synthesis of theranostic nanocomposite using drug intercalation process.

Herein, we synthesized theranostic nanoparticles by Gd doping onto Zn/Al-LDH. Gallic acid was first intercalated into the interlayers of the LDH-Gd and AuNPs were then grown on the surface of the LDH nanoparticles. The LDH prepared via co-precipitation method was used as the nanocarrier, while Gd and AuNPs were used as the main contrast agent and booster for MRI, respectively.

## 2. Results and Discussion

**Figure 1:**
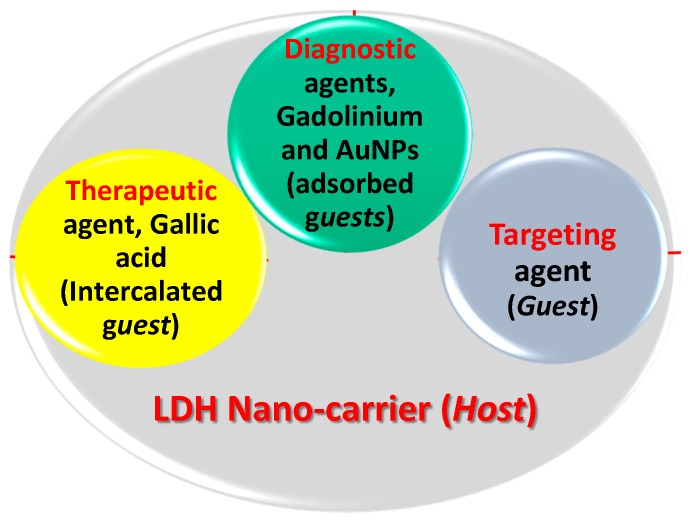
Schematic arrangement of GAGZAu nanocomposite in respect to theranostic delivery system in a typical host–guest relationship.

The final GAGZAu nanoparticles were subjected to various characterizations as will be reported later, although the analyses were done at every step of the synthesis, starting with the LDH nanocarrier itself. Figure 1 is a representative of a typical multimodal theranostic setting, similar to a host–guest reaction in supramolecular chemistry, where a nano-carrier, a 2D host was first loaded with therapeutic agent (the first guest) by the intercalation process, gallic acid. Following the formation of pure phase, diagnostic agents (the second guests), Gd, and AuNPs were loaded. A third guest, a targeting agent, can be also loaded, resulting in the formation of a multimodal theranostic nanodelivery system [8]. However, the loading of a targeting agent will be done in our near future work. The mechanism of bonding between the LDH and the GA is via hydrogen bonding due the surplus OH groups in the anionic guest as well as ion exchange with the interlayer anions [20]. Whereas the contrast agents are bonded to the LDH through van der Waals forces of attraction.

### 2.1. X-ray Diffraction 

**Figure 2:**
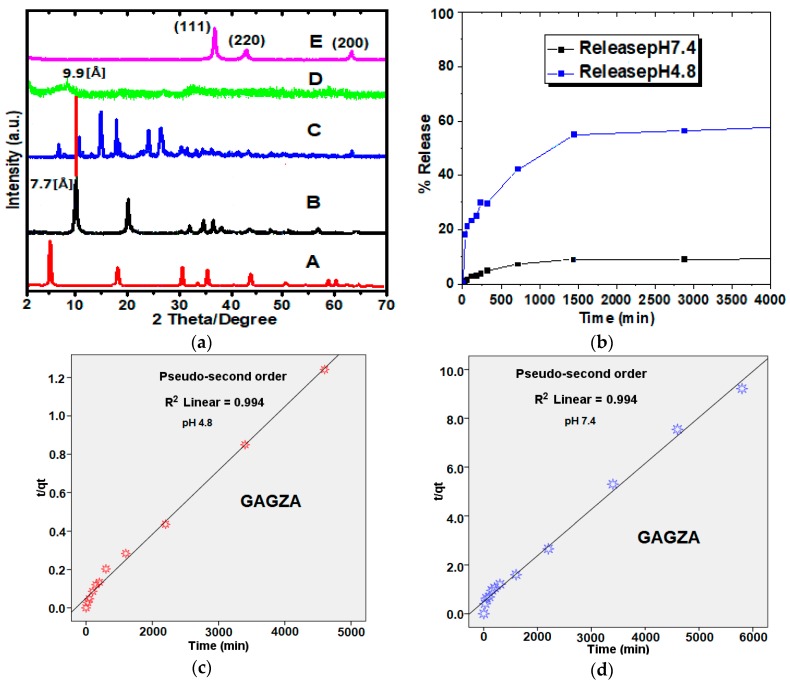
(**a**) X-ray diffractograms of (A) Gd(NO_3_)_3_, (B) Zn/Al-LDH, (C) pure gallic acid, (D) gallic acid-Zn/Al-LDH-Gd nanocomposite (GAGZA), (E) gallic acid-Zn/Al-LDH/Gd-Au nanocomposite (GAGZAu); (**b**) Release profiles of gallic acid intercalated into Zn/Al-LDH-Gd nanoparticles (GAGZA) in phosphate-buffered solutions at pH 4.8 and pH 7.4; (**c**) Data of gallic acid released from GAGZA obtained at pH 4.8 fitted to pseudo-seconder order; (**d**) Data of gallic acid released from GAGZA obtained at 7.4 fitted to pseudo-seconder order.

The diffractograms in Figure 2a indicate various patterns of the different stages of the nanocomposite synthesis, from the starting material to the final nanocomposite (A–E respectively). The diffractogram (A) represents the Gd(NO_3_)_3_, (B) represents LDH, (C) is for gallic acid, which are all in a pristine state. Further, the pattern of GAGZA (D) represents the first stage of the formation of theranostic nanocomposite, that is, the anticancer drug was intercalated into the LDH/Gd (A) interlayers at this stage. This as a result shows increase in basal spacing up to 9.9 Å, that is much higher than 7.7 Å of the LDH basal spacing; which strongly indicates the drug intercalation had taken place. In addition, the slight shift to a lower 2θ angle also implies the intercalation of the therapeutic agent GA into the interlayers of the LDH has taken place. The diffractogram of the theranostic GAGZAu (E) nanoparticles however, did not indicate most of the reflections of the LDH. This is presumably due to the surface coating of the AuNPs on the surface of the theranostic nanoparticles. Nonetheless, the pattern (Pattern 4-784) observed match with FCC (111, 200, and 220) of pure AuNPs [21].

### 2.2. Drug Release Profile and Kinetics from GAGZA Nanocomposite 

The amount of drug loaded and in vitro drug release in the nanoparticles was calculated using a standard curve of the UV–Vis spectrometer at λ_max_ of the drug, 264 nm. As seen in Figure 2b, the maximum percentage of about 60% of the drug is released over a period of 5000 min (84 h) with 50% drug loading. The release was done using PBS buffer solution at pH 4.8 and 7.4. The release in the slightly alkaline pH is observed to be significantly lower (10%) than in the acidic pH (60%); which could be attributed to ion exchange occurring in the buffer media between the anions in the drug and the LDH nanocarrier [22]. Nonetheless, the sustained release profiles suggest safe release of the drug from the nanocarrier to the solution under acidity of pH 4.8 and less release at the higher pH 7.4. The huge difference in the release pattern of the pH is attributed to the mechanism or behavior of the nanocarrier in buffer solutions, as it is a common attribute of LDH interlayers to dissolve in acidic media together with the anionic guest on the external surface of the LDH [15], hence resulting in more release of the intercalated guest. This indicates the suitability of the nanohybrid as a good candidate for anticancer research, as rightly reported in literature where LDH nanoparticles was reported to have penetrated the cervical cancer cells via mediated endocytosis [23]. Furthermore, to understand the mechanism of the drug release, the data from the release study was fitted to three different kinetics models, namely

(1) $$\text{Pseudo-first order:}\;\ln(q_{e}-q_{t})\;=\;\ln q_{t}\;-\;kt$$

(2) Pseudo-second order: tqt = 1kqe2 + tq

(3) $$\text{Parabolic diffusion:}\;\left(1-M_{t}/M_{o}\right)/t\;=\;kt^{-0.5}\;+\;b$$

The parameters used in the fitting equations are the drug content in the nanocarrier during release which are represented as $M_{t}\;$ and $M_{o}$, the amount released at equilibrium and time t designated as $q_{e\;}$ and $q_{t}$ respectively; and the release constant k [15].

The three models as represented in Equations (1)–(3), gave different kinetic processes, the model that best describes the kinetic release of the gallic acid from the nanocomposite at both pH (7.4 and 4.8) is the pseudo-second order model, as expressed in Equation (2). The parameter tqt is plotted against the time t, which gave correlation coefficients $\left(R^{2}\right)$ of 0.994 at both pH 7.4 and 4.8 (Figure 2c,d). The rate constants (k) were determined to be 4.7 × $\;10^{-2}$ and 8.5 × $\;10^{-3}$ g/mg h, for pH 7.4 and 4.8, respectively. These results are consistent with other reported findings on drug release using LDH nanocarriers [15] and other nanocarriers [24]. Table 1 is a compilation of the fitted data of correlation coefficients $\left(R^{2}\right)$, rate constants (*k*), and half-lives $\left(t_{1/2}\right)$, as well as saturation points (%) of gallic acid released from GAGZA at pH 7.4 and 4.8.

### 2.3. Surface Morphology and Elemental Content Analysis 

**Figure 3:**
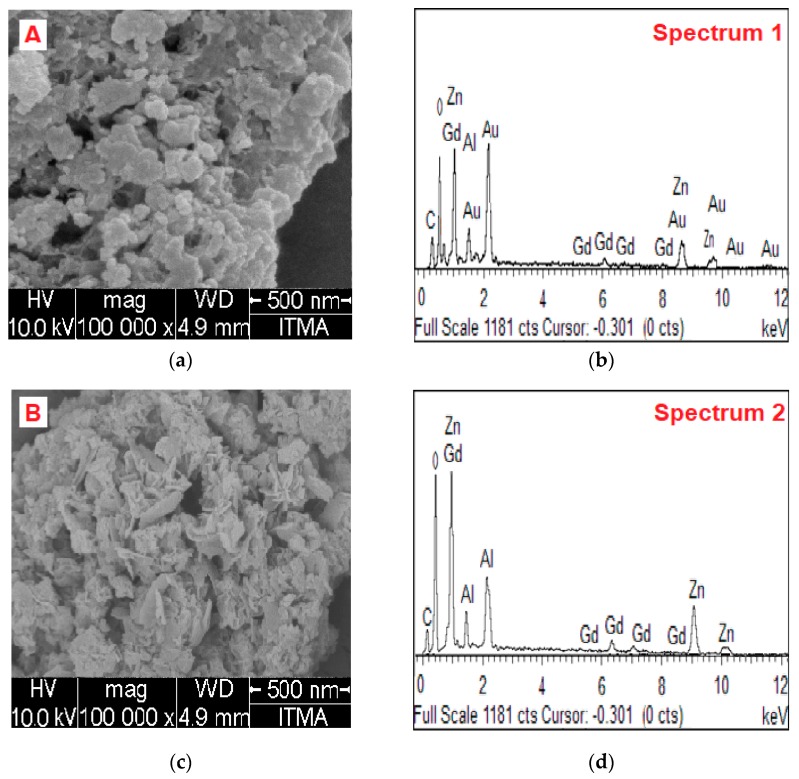
(**a**) Field emission scanning electron microscopy (FESEM) micrograph (A) and (**b**) Energy dispersive X-ray (EDX) spectrum (1) of gallic acid intercalated in LDH/Gd nanoparticles coated with AuNPs (GAGZAu); (**c**) micrograph (B); and (**d**) EDX spectrum (2) of gallic acid intercalated in LDH/Gd nanoparticles (GAGZA).

The morphology of the nanoparticles was studied before and after the addition of AuNPs, that is for GAGZA and GAGZAu. Figure 3a depicts micrographs of GAGZAu and Figure 3c shows GAGZA micrographs. Though evenly distributed, the nanoparticles are observed to be agglomerated at dry state. The agglomeration may be due to surface energy, a phenomenon associated with small sized nanoparticles [25]. As observed in Figure 3a, the AuNPs coated on the GAGZA nanocomposite appears to have covered the LDH structure which has affected the thermal stability of the final GAGZAu nanocomposite as discussed in the TGA analysis chapter, as well as the MRI contrast due to increased surface area to volume ratio in the nanohybrid. The FESEM micrographs of the nanoparticles analyzed with EDX indicated the presence of the all the active elements in the nanoparticles (Zn, Al, O, C, Gd, and Au). 

The EDX spectra were generated via random capture of the whole area of the FESEM micrographs attached to the spectra. As anticipated, the elements contained in spectrum 1 (Figure 3b) obtained from GAGZAu contain various reflections of Au which appeared to dominate the Gd reflections in spectrum 2 (Figure 3d), indicting the coating of the LDH surface with AuNPs. The few reflections of Gd are also suggesting the presence of the element in the micrograph. However, as expected also, the spectrum 2 obtained from GAGZA micrograph, before the addition of AuNPs (Figure 3d) showed dominant reflections from Gd with no sign of Au signals, which confirms the initial formation of LDH-Gd nanocomposites. Likewise, the Zn and Al reflections which appeared in both spectra confirm the structure of Zn/Al-Gd LDH. Further, aside from the C and O signals that appeared in the spectra, which are likely from the intercalated GA drug, no other reflections are observed. This shows the high percentage of purity of the nanocomposite at various stages of synthesis. The results appear to be in conformity with ICP-ES elemental composition analysis carried out on the samples, which showed presence of all the intended elements in the nanocomposite. 

### 2.4. Size, Shape, and Distribution Analysis

**Figure 4:**
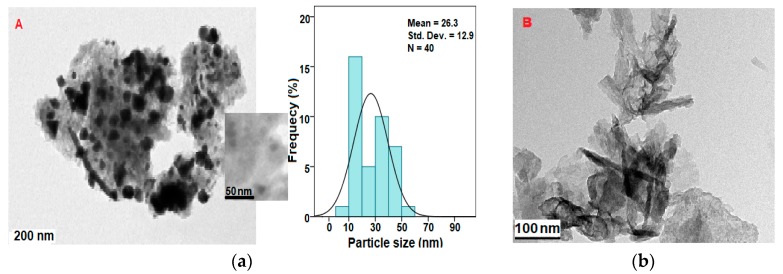
(**a**) Transmission electron microscopy (TEM) micrograph (A) and particle size distribution of gallic acid-intercalated in Zn/Al-LDH/Gd nanoparticles coated with AuNPs (GAGZAu) and micrograph (B); (**b**) of gallic acid intercalated in Zn/Al-LDH/Gd nanoparticles (GAGZA).

TEM was used to analyze size and shape/distribution of the nanoparticles. The GAGZA micrograph (Figure 4b) indicates various sizes of the nanoparticles in different shapes, including rod-shaped nanoparticles which are conspicuously seen throughout the micrographs at different magnifications. All the nanoparticles are found to be within the nanorange (1–100 nm) [1]. 

The micrograph of the sample containing AuNPs (GAGZAu) (Figure 4a) evidently indicates the presence of the AuNPs in various sizes and shapes—such as hexagonal, triangular, and spherical shapes—thus confirming the growth of the AuNPs as indicated by other characterization results of the sample. The mean size of the AuNPs is approximately 26.3 nm as indicated in the histogram in Figure 4a, which are predominantly evenly distributed on the surface of the LDH nanocomposite across all shapes and sizes. 

### 2.5. Chemical Interaction Studies (FT-IR Analysis)

**Figure 5:**
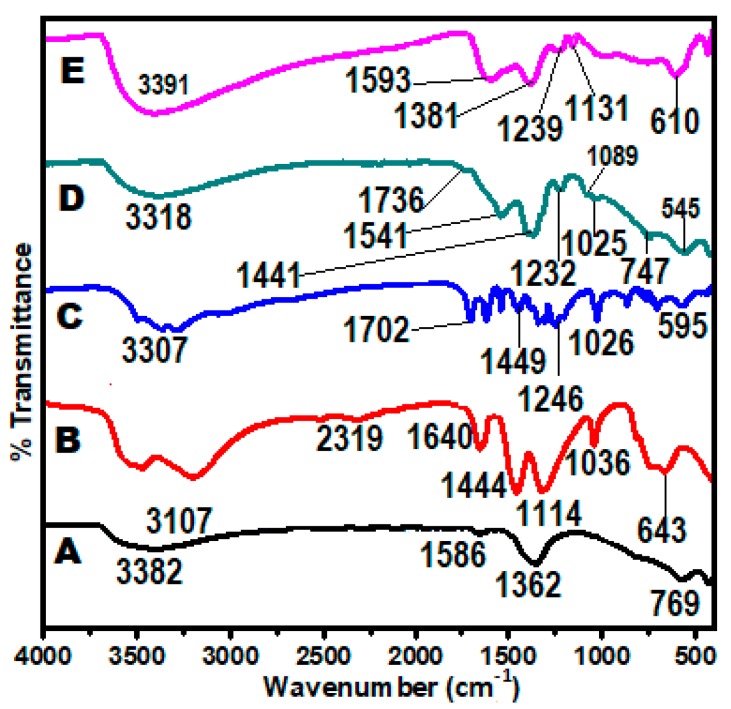
Fourier transform infrared spectroscopy (FT-IR) spectra (A) LDH, (B) Gd(NO_3_)_3_, (C) pure gallic acid, (D) gallic acid-Zn/Al-LDH/Gd nanoparticles (GAGZA), (E) Gallic acid-Zn/Al-LDH/Gd-Au nanoparticles (GAGZAu).

The chemical interactions and changes between the nanocarriers and nanoparticles were studied using FT-IR spectroscopy. The changes in functional groups, chemical bonds and shifts in wavenumbers are the indication that some of the interactions have taken place. For easy comparison, the analysis was done on the pristine samples and the nanoparticles. Figure 5 depicts the FT-IR spectra of the Zn/Al-LDH, Gd(NO_3_)_3_, GA, GAGZA, and GAGZAu (A–E). The bands between 3307–3391 ${\mathrm{cm}}^{-1}$ which appear in all the FT-IR spectra are associated with OH stretching vibrations [26]; 1736 and 1702 ${\mathrm{cm}}^{-1}$ are characteristic absorptions of C=O; C=C aromatic ring stretching modes are represented by the bands 1541 and 1441, and 1541 and 1449 ${\mathrm{cm}}^{-1}$ in the GAGZA and GA spectra respectively; the band at 595 $\;{\mathrm{cm}}^{-1}$ represents bending vibration of OH carboxylic group. The bands at 1232, 1089, and 1025 ${\mathrm{cm}}^{-1}$ which appear in the GAGZA spectrum and at 1246 and 1026 ${\mathrm{cm}}^{-1}$ in the GA spectrum, are of C–O stretching vibrations [27]. The nitrate group stretching mode appears as a band at 1384 ${\mathrm{cm}}^{-1}$ of the Zn/Al spectrum; the band is noticeably absent in all other spectra; this is due to the loss of the nitrate anion in the bonding process, while $H_{2}$ bending vibration band can be seen at 1640 ${\mathrm{cm}}^{-1}$. Furthermore, as expected the spectra of GAGZAu (E) appeared to be similar to spectra D of GAGZA, with the only difference is blue-shift in the band positions to higher wavenumbers. Generally, there are all shifts in the absorptions of the bands which are as a result of the bonding that took place between the guests and the LDH host at different stages of synthesis.

### 2.6. Thermal Stability Analysis (TGA/DTG)

**Figure 6:**
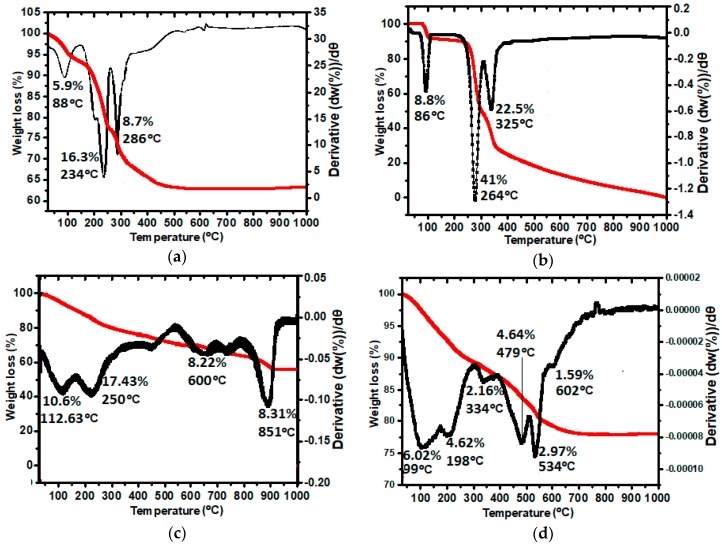
(**a**) Thermogravimetric and differential thermogravimetric analyses (TGA/DTG) thermograms of pure LDH; (**b**) pure gallic acid; (**c**) gallic acid-LDH/Gd nanoparticles (GAGZA); (**d**) gallic acid-LDH/Gd-Au nanoparticles (GAGZAu).

The thermal stability of the nanoparticles and the pristine materials (GA and LDH) was tested with thermogravimetric and differential thermogravimetric analyses (TGA/DTG). To have more understanding of the thermal changes that occur, the TGA/DTG patterns of Zn/Al-LDH, GA, GAGZA, and GAGZAu were obtained as shown in Figure 6, and some key parameters have been highlighted in Table 2, which are: maximum peak temperature (Tmax), decomposition temperature range (Trange), and change in mass [(decomposed mass) Delta m] [28]. The peak at 88 °C which corresponds with weight loss of 5.9% in the Zn/Al-LDH thermogram (Figure 6a) is representative of physisorbed water loss on the surface and interlayer of the LDH.

The second peak at 243 °C with 16.3% mass loss and the third peak with 8.7% weight loss are reflective of nitrate decomposition and metal hydroxide layers dehydroxylation. The GA thermogram in Figure 6b shows three significant peaks, the first peak at 86 °C with 8.8% weight loss is attributed to absorbed water, while the peak at 264 °C which accounts to substantial weight loss of 41.0% is due to the dihydroxylation and decomposition of gallic acid structure, and lastly the one at 325 °C (22.5%) which represents residue decomposition. 

Figure 6c depicts the thermogram of the GAGZA nanoparticles. The peak at 112 °C which represents 10.6% weight loss is related to removal of water. The peak next to it at 250 °C representing 17.4% loss of weight is due to dehydroxylation of the intercalated gallic acid as well as partial decomposition of nitrate. The peak at 600 °C indicating 8.2% weight loss could be attributed to the decomposition of Gd(NO_3_)_3_ together with the nitrate ions of LDH layers in the nanocomposite. The sharp peak at 851 °C (8.3%) indicates the collapse of hydrotalcite-like layers and complete decomposition of the nanoparticles [29], which shows improved thermal stability that took place in comparison with the individual components. This is due to electrostatic attraction bonding that occurred between the negatively charged gallic acid functional groups and the positively charged LDH interlayer surfaces. Interestingly, the thermogram of the final nanocomposite which is the gold coated GAGZAu (Figure 6d), showed less thermal stability compared to the GAGZA thermogram. For instance, the first two peaks (99 and 198 °C) which represent loss of water and that of dihydroxylation respectively, appear to be at lower temperatures in comparison with GAGZA composite (112 and 250 °C). The peak at 602 °C and 1.59% weight loss of GAGZAu is associated with nitrate ions decomposition in nanocomposite. The peak associated with AuNPs surface decomposition is at 334 °C [30], amounting to about 2.2% weight loss. Generally however, the nanohybrid appear to be less thermally stable after coating with the AuNPs as depicted in Table 2. Nonetheless, the AuNPs could be more thermally stable when synthesized separately in their pure nanoparticles form using capping agents. As reported in literature, thermal stability of AuNPs depends on certain factors, such as the capping or stabilizing agents used in the synthesis and the type of bonding that occur between them determine the thermal stability, thus, weak bonds result in less stability and vice versa [31,32,33,34]. In our case, the reduction in thermal stability after AuNPs coating is presumably due to weak interaction between the AuNPs and the LDH nanocomposite surface, since no capping agent was used for the AuNPs synthesis. 

### 2.7. CHNS-ICP-ES Analyses

The composition of the GAGZAu nanocomposite was determined by combining CHNS and ICP-ES analyses, the individual data acquired were used to estimate the percentage content of each element. As indicated in Table 3, the constituents of the nanocomposite have, as obtained from the results, indicated successful intercalation of the guest drug (gallic acid) into LDH interlayer gallery. Additionally, the mole ratio of the ${\mathrm{Zn}}^{2+}$/${\mathrm{Al}}^{3+}$ in the GAGZAu nanocomposite and in the LDH was observed to be the same (0.1%). The ICP-ES data also shows presence of Gd and Au, indicating successful integration of Gd in the LDH-Gd block as well as coating of the surface with AuNPs. Moreover, the results are a validation of the EDX results reported earlier in this paper, which similarly confirms the presence of the aforesaid elements in the nanocarrier. 

### 2.8. In Vitro Cytotoxicity Studies against HepG2 and 3T3 Cell Lines

**Figure 7:**
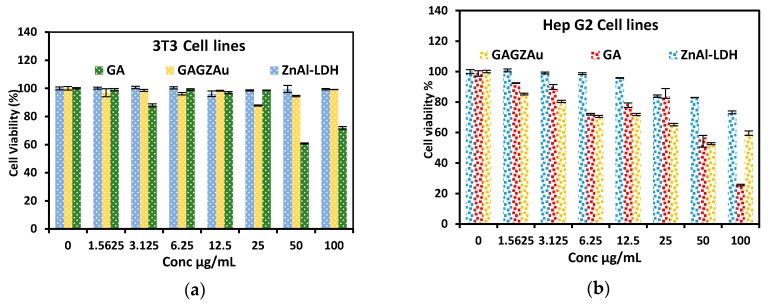
Cytotoxicity study of GAGZAu, LDH, and free drug at various concentrations exposed to (**a**) cancer cell lines (HepG2) and; (**b**) normal cell lines (3T3).

The toxicities of the GAGZAu, the free drug and the nanocarrier were tested via cell viability study against liver cancer cell lines and normal human cell lines, HepG2 and 3T3, respectively. The cell lines were exposed to different concentrations of the aforementioned samples, starting with 0 (control) to 100 μ/mL. As observed in Figure 7, the free drug, the LDH and the GAGZAu nanohybrid tested against the 3T3 cell lines indicate nearly 100% cell viability in most of the concentrations. The 50 and 100 μ/mL dosage of the free drug however, shows slightly lower viability. This is expected as anticancer drugs are known to be cytotoxic and have certain undesirable side effects on normal cells [35,36]. Likewise, Gd-based contrast agent has been reported to have caused nephrogenic systemic fibrosis, which is associated with the toxicity of Gd [37,38]. Nevertheless, such toxicities are reduced when the anticancer agents and other agents are intercalated into LDH nanocarriers [39] or adsorbed on the surface, as observed in our case, the toxicities of GA and Gd have been virtually neutralized in the nanoparticles form, that is in the GAGZAu tested for the normal cell lines. 

The HepG2 cell lines were similarly treated with different concentrations of the free drug, LDH and GAGZAu. Although the toxicity of the gallic acid against the HepG2 cell lines decreased in the nanoparticles form, the viability of the cells treated with GAGZAu still indicates cytotoxicity against HepG2 cell lines. Moreover, the LDH nanocarrier affects the therapeutic activity of the gallic acid; the nanoparticles could be more cytotoxic to other cancer cell lines. Nonetheless, the cells treated at 50 μ/mL of GAGZAu show the highest anticancer activity, which is due to higher release of the anticancer compounds contained inside the nanocomposite. Even though both 3T3 and HepG2 cells lines are non-phagocytic and have negatively charged cell surfaces, only the cancer cell lines will be susceptible to anticancer activity effects of the nanocomposite. The general mechanism of GA uptake by the cells is through electrostatic attraction which is prompted by cellular endocytosis between the positively charged GAGZAu surface and the negatively charged cell surface [20]. The individual specific mechanism through which the GAGZAu intake occurs in the 3T3 and HepG2 cannot be described via simple MTT assay alone, other more precise experiments such as flow cytometry have to be applied.

### 2.9. Magnetic Resonance Imaging

**Figure 8:**
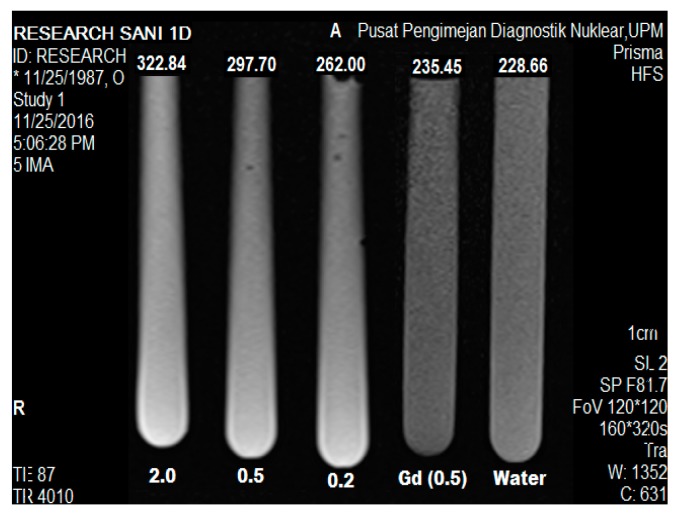
GAGZAu image at different ${\mathrm{Gd}}^{3+}$ concentrations (2.0, 0.5, 0.2), Gd (0.5) *w*/*v* and water taken from magnetic resonance imaging MRI Prisma 3-Tesla machine.

The magnetic resonance image of the samples was captured as shown in Figure 8, where five tubes were arranged, each representing GAGZAu nanoparticle solutions at different concentrations in order of increasing ${\mathrm{Gd}}^{3+}$ concentration; 0.2, 0.5, and 2.0 *w*/*v*, Gd(NO_3_)_3_ and distilled water (as the reference). The average intensity of each tube was measured using Syngovia MRI software, and signal mean intensities were calculated. The signals intensities of each concentrate tube was documented to increase steadily and proportionally as the concentration of ${\mathrm{Gd}}^{3+}$ in the nanohybrid, that is 322.44, 297.70, and 262.00 for 2.0, 0.5, and 0.2 *w*/*v* concentration, respectively. In addition, the R1 signal intensity of GAGZAu at the lowest dosage (0.2 *w*/*v*) indicates higher R1 signal intensity (262.00) than Gd(NO_3_)_3_ (235.45) at 0.5 concentration and the water reference (228.66). This is indicative that R1 signal increase is as a result of the formation of Gd-based nanoparticles. The boost occurred as a result of the shortening of the longitudinal relaxation time (T1) [40,41] due higher surface area to volume ratio of AuNPs coated on the surface of the nanoparticles, which improves water solubility [41] and molecule movement, and proton exchange within the ${\mathrm{Gd}}^{3+}$/LDH lattice [42,43]. The phenomenon is suspected to be connected with the change in structure of the nanohybrid after AuNPs surface coating, as observed in the GAGZAu TEM and FESEM micrographs. The free movement of water molecules through the nanohybrid results in shortening of the longitudinal relaxation time as well as reduces short circulation in the system, which in turn increases the R1 value of the recorded MR image. The longitudinal relaxation time as stated earlier in this work is responsible for the contrast effect of MR imaging [44]. Similar outcome was reported by previous works done using Gd/LDH-Au nanoparticles [8].

## 3. Materials and Methods 

### 3.1. Materials

Gallic acid with molecular weight of 170.12 g/mol and 98% purity, sodium hydroxide molecular weight of 40.00 g/mol and 98% purity and phosphate-buffered saline were purchased from Sigma-Aldrich (St. Louis, MO, USA). Zinc nitrate hexahydrate molecular weight of 297.47 g/mol, 98% purity, and aluminium nitrate hexahydrate molecular weight of 375.13 g/mol, 98% purity, were purchased from Systerm ChemPur (Shah Alam, Selangor Darul Ehsan, Malaysia). Gadolinium (III) nitrate hexahydrate and molecular weight of 451.4 g/mol with 99.9% purity and tetrachloroauric(III) acid trihydrate, 393.83 g/mol and 49% Au purity were purchased from Acros Organics (Morris Plains, NJ, USA). Sodium borohydride molecular weight of 37.83 g/mol, and 99% purity was purchased from Fluka Analytical (St. Gallen, Switzerland). All chemicals were used as received without further purification. Deionized water was used throughout the experiment.

### 3.2. Synthesis of Gd-Zn/Al-Layered Double Hydroxide 

Zn/Al-layered double hydroxide was synthesized using Zn(NO_3_)_2_ and Al(NO_3_)_3_ at a molar ratio of 4:1 of ${\mathrm{Zn}}^{2+}$ to ${\mathrm{Al}}^{3+}$. Gd(NO_3_)_3_ (0.0008 M) was firstly dissolved in 250 mL deionized water before the addition of the Zn/Al nitrate salts. Dropwise addition of NaOH (2 M) was immediately followed until a pH of 7 was reached. The synthesis was conducted under nitrogen flow and vigorous stirring. The slurry obtained at the end of the process was aged for 18 h at 70 °C, centrifuged, washed with deionized water (three times), and oven dried at 60 °C.

### 3.3. Loading of Gallic Acid into Gd-Zn/Al-LDH Nanoparticles (GAGZA)

Briefly, gallic acid, 0.2 M solution was prepared by dissolving 3.6 g of the drug into 50 mL deionized water while stirring and heating at 45 °C. Under continuous nitrogen flow with vigorous stirring, the drug solution was simultaneously added dropwise with NaOH (2 M) into the 250 mL solution of Gd-Zn/Al-layered double hydroxide. The solution was kept until pH 7 was reached and the drug was completely loaded. The mixture was then aged for 18 h at approximately 70 °C. The resultant slurry obtained was filtered, washed/centrifuged using deionized water, and oven dried at 60 °C for 12 h.

### 3.4. Doping of Gold Nanoparticles (AuNPs) onto Zn/Al-Gd GA LDH (GAGZAu)

Appropriate amount of GAGZA was ultrasonically dispersed for 5 min, under gentle stirring; 2% HAuCl_4_ (6 mL) solution was added. After 5 min stirring, 0.125 M NaOH (4 mL) was added; the dispersion was allowed to stir for 5 min before the temperature of the solution was raised to 60 °C and stirred for 24 h in dark conditions. The resulting precipitate was obtained after centrifuge/filtration, the mixture was re-dispersed in 30 mL deionized water; 1 M NaBH_4_ (20 mL) was added and stirred for an hour. The final suspension obtained was washed six times using a centrifuge, filtered, and dried at 70 °C in an oven. 

### 3.5. Characterization

An XRD-6000 (Shimadzu, Tokyo, Japan) X-ray diffraction (XRD) instrument was used for crystallographic analysis of the powdered samples. CuK_α_ radiation (λ = 1.5418 Å) at scan speed of 4°/min, using a range of 2–70 °C at 30 kV and 30 mA was used in obtaining the XRD patterns. A ultraviolet–visible (UV–Vis) spectrophotometer (PerkinElmer, Singapore) (Lambda35) was used for the controlled release and optical property studies. Fourier transform infrared (FT-IR) spectra of the materials were obtained using a Thermo Nicolet, Nicolet 6700 model (Thermo Scientific, Waltham, MA, USA). The spectra were obtained using the potassium bromide (KBr) discs at 10 ton pellet pressing and a resolution of $4{\;\mathrm{cm}}^{-1}$ over a range of 400–4000 ${\mathrm{cm}}^{-1}$. A PerkinElmer spectrophotometer (PerkinElmer, Wellesley, MA, USA) (model Optima2000DV) inductively coupled plasma atomic emission spectrometry (ICP-ES) was employed in studying the composition of zinc, aluminum, gadolinium, and gold content of the nanoparticles. A CHNS-932 LECO (LECO, St. Joseph, Michigan, USA) instrument was used in determining carbon, hydrogen, nitrogen, and sulphur (CHNS) content in the sample. A Mettler-Toledo instrument (METTLER TOLEDO, Shah Alam, Selangor, Malaysia) was used for thermogravimetric and differential thermogravimetric (TGA/DTG) analyses. A sample heating rate of 10 °C/min and a range of 20–1000 °C were used in this work. The analysis was carried out at a continuous nitrogen flow at 50 mL/min flow rate. High resolution transmission electron microscope (Hi-TEM) (FEI Tecnai TF20 X-Twin, Hillsboro, OR, USA) and FEI Nova NanoSEM 230 field emission scanning electron microscope (FESEM) (FEI, Hillsboro, OR, USA) were employed for shape/sizes analyses and morphological analyses, respectively while energy dispersive X-ray spectroscopy (EDX) (FEI, Hillsboro, OR, USA) was used for elemental analysis of the FESEM micrographs. 

### 3.6. Drug Release Study

As mentioned earlier, gallic acid release from GAGZA was studied using a PerkinElmer Lambda 35 UV-Vis spectrophotometer (PerkinElmer, Singapore). Firstly, standard solutions of gallic acid at different concentrations were prepared. Appropriate amount of GAGZA was dissolved in 5 mL of HCl (1 mol/L) and then diluted with 45 mL deionized water. The lambda max of the drug in the solution was found to be 264 nm, which was used in determining the standard curve for the standard solutions and drug loading capacity of GA. Kinetic release of the loaded GA from GAGZA was done at pH 7.4 and 4.8 in a phosphate-buffered solution (PBS). Briefly, 25 mg of the sample was dispersed in 30 mL of the PBS in tubes. The tubes were placed in an oil bath shaker at 37 °C and 3 mL of the solutions were withdrawn and replaced with 3 mL of pure PBS at time intervals of 0.5, 1.0, 2.0, 3.0, 4.0, 5.0, 6.0, 12.0, 24.0, 48.0, 72.0, 92.0, and 120.0 h. The release media extracted were analyzed at lambda max = 264 nm wavelength with a UV–Vis spectrophotometer (PerkinElmer, Singapore).

### 3.7. Cell Culture

The cell lines used for cancer study was HepG2 (human liver hepatocellular carcinoma cell line) and normal cell lines, 3T3 (standard fibroblast cell line) for toxicity study, which were obtained from ATCC. RPMI 1640 was used as a medium for cell lines growth, which contains 1% penicillin/streptomycin and 10% fetal bovine serum (FBS). Cells culture was done at approximately 80% confluence, as adherent monolayers. Temperature was set at 37 °C and in a 5% ${\;\mathrm{CO}}_{2}$ humidified atmosphere. Cell harvest was done via trypsinization (in brief) with trypsin-EDTA solution. All reagents are research grade and were used as received.

### 3.8. MTT Cell Viability Assays

RPMI 1640 was also used as a medium for the cancer cell lines and normal cells, which were grown in a humidified incubator at 37 °C and 5% ${\mathrm{CO}}_{2}$. The cells were grown and were harvested and counted. Prior to 24 h incubation, the cells were transferred to 96-well plates (1 × 104 cells/well) and then the GAGZAu nanoparticles, LDH, and free GA were added. The medium was kept for 24 h to allow the cells to attach to the surface before treatment. The cells containing the GAGZAu, Zn/Al-LDH, and free drug were administered and applied in different concentrations prior to treatment and 72 h incubation. For MTT, 5 mg of (3-[4,5-dimethylthiazol-2-yl]-2, 5-diphenyltetrazolium bromide was dissolved in PBS (2 mL). To each of the 96-well plates, 20 μL of the MTT solution was added and incubated at 37 °C for 3 h until formazan product was developed (purple-colored). Suction method was employed to remove the solution in each well containing media, unbound MTT, and dead cells; 100 μL of DMSO was added to each well. The optical densities of the cells were obtained with the use of a microplate reader at 570 nm. Prior to the measurements, the cells were shaken. All analyses were done in triplicate and the cell viabilities/increase was presented in percentage in reference to control cells. 

### 3.9. MR Imaging Analysis

The GAGZAu nanocomposite was tested for MRI signal intensity using 3.0 T MRI clinical instrument (3.0 T Siemens Magnetom, Erlangen, Germany). Prior to the analysis, GAGZAu was prepared in various concentrations (2.0, 0.5, and 0.2 *w*/*v*) according ${\mathrm{Gd}}^{3+}$ concentration. Gd(NO_3_)_3_ (0.5 *w*/*v*) and water together with samples were then placed in a 1 mL tube. The MR image was acquired by attaching the tubes to an MRI phantom, and then the phantom was placed in the instrument. The T1-weighted images of the samples were captured at TR/TE: (83/9000) 224 × 220 s, field of view (FOV): 120 × 120. The MR image was analyzed and signal intensities of the individual samples were extracted with Syngovia (MRI and CT reporting software, syngo MR E11, Siemens, Erlangen, Germany, 2013).

## 4. Conclusions

Gd-based nanocomposite (GAGZAu) was developed in this work, which was painstakingly analyzed to understand and ascertain its theranostic properties. The results obtained from different stages of the nanoparticles synthesis until the final nanocomposite GAGZAu shows the potentiality of the nanohybrid to be used as a theranostic bimodal delivery agent. The in vitro drug release study shows higher drug release in pH 4.8 (pH of cancer cells), indicating the capability of the platform to convey the GA into cancer cells and prevent premature release in the blood stream. Moreover, the nanocomposite shows reasonable cytotoxicity to HepG2 cancer cell lines and negligible toxicity to 3T3 normal cell lines. The preliminary MR imaging analysis conducted on the developed theranostic nanohydrid also indicates improved MRI contrast of T1-weighted image obtained as compared to pure Gd(NO_3_)_3_ and water. Based on the promising outcome of this work, further works such as in vivo testing could be done on the GAGZAu nanocomposite, which would improve its theranostic prospects, such as reducing artefact formations like short circulation and tissue specificity that are associated with gadolinium-based contrast agents as well as toxicities of chemotherapeutic agents. 

## Figures and Tables

**Table 1:** Fitted data of correlation coefficients ($R^{2}$), rate constants (*k*), and half-lives ($t_{1/2}$) of gallic acid released from GAGZA at pH 7.4 and 4.8.

Medium pH	Release Saturation (%)	$\left(R^{2}\right)$	$\left(R^{2}\right)$	$\left(R^{2}\right)$	Rate Constant (k)	$t_{1/2}\;$ (min)
		Pseudo-First Order	Pseudo-Second Order	Parabolic Diffusion		
7.4	18	0.432	0.994	0	4.7×$10^{-2}$	520
4.8	62	0.094	0.994	0.7	8.5×$10^{-3}$	400

**Table 2:** Maximum peak temperature (Tmax) decomposition temperature range (Trange) and change in mass (Delta m).

Sample	Tmax (°C)	Trange (°C)	Delta m (%)
(A) LDH	234	88–286	30.9
(B) Gallic acid	264	86–325	72.3
(C) GAGZA	851	112–851	44.6
(D) GAGZAu	534	99–602	22.2

**Table 3:** Elemental analysis results for Zn/Al-layered double hydroxide (LDH) nanocarrier and GAGZAu nanocomposite.

Sample	C% ^•^	H% ^•^	N% ^•^	Zn% ^••^	Al% ^••^	Gd% ^••^	Au% ^••^	$Zn^{2+}$/$Al^{3+}$% ^••^	Drug% ^•^
Zn/Al-LDH	-	-	7.3	45	5.2	-	-	0.1	-
GAGZA-Au	8.714	2.186	0.4208	8.82	1.02	1.56	3.0	0.1	50

^•^ Calculated from CHNS analysis; ^••^ Calculated from ICP-ES analysis.
